# Supplementary material for: Motivation, barriers and preferences of lifestyle changes among older adults with frailty and mild cognitive impairments: A scoping review of qualitative analysis
Source: PLoS One. 2025 Jan 20;20(1):e0314100. doi: 10.1371/journal.pone.0314100 (PMC11745415; doi:10.1371/journal.pone.0314100)
Supplement: Attachment — Submitted filename: Response to Reviewers.docx [file pone.0314100.s003.docx]

| Reviewer comment | Response | Location in Manuscript |
| --- | --- | --- |
| Reviewer 1 | | |
| Introduction |  |  |
| 1. It is necessary to better problematize the relevance of analyzing the effect of lifestyles on physical frailty and cognitive impairment in older people and the relevance of the review on the barriers and motivations of older people to participate in interventions. | Thank you for your consideration in reviewing this manuscript and the comments given are highly appreciated to improve the writing.  We have refined the introduction section to better emphasize the importance of analysing the effect of lifestyle intervention on both physical frailty and cognitive impairment in older adults.  We have also clarified the relevance of identifying barriers, motivators and preferences regarding participation in lifestyle interventions particularly in populations with physical frailty and mild cognitive impairments. This has been done to align with the objective of our review. | Introduction section  Page 3 and 4 |
| 2. It would be necessary to define what is understood as lifestyle and lifestyle intervention. A better problematization is necessary and the ultimate goal is to identify the evidence on this topic. | We appreciate this suggestion and have added a description of lifestyle intervention in Introduction section.  Additionally, a more detailed explanation of lifestyle intervention has been included in the methodology section. | Page 3  Line 59-63  Page 7  Line 202-204 |
| Method |  |  |
| 3. Regarding stage 3: regarding the inclusion criteria, it is necessary to include whether the year of publication was used as a criterion. | Thank you for the suggestion.  The year of publication was mentioned under the method section, under sub heading Stage 2: Identifying Relevant Studies. The search limit has been set to studies published between January 2010 to October 2023.  We have included the year of publication inclusion criteria in the stage 3 sub section. | Page 6  Line 187-188  Page 7  Line 200-201 |
| 4. It is important to describe the scope and limitation of the use of Rayyan Ai software for the selection of evidence.   Explain better what tasks the software performs, I am concerned that the review is biased by the software and is not a review carried out by the authors. | We are grateful for your feedback on this matter.  To clarify, Rayyan AI was employed only as an organisational tool to assist with managing references, detecting duplicates and facilitating title and abstract screening. However, all inclusion and exclusion decisions were made manually by the authors through independent screening and consensus meetings. We have updated the manuscript to reflect this and assure the reviewer that the software did not influence the final decision-making process.  We have included a description of the usage of Rayyan AI in the method section to clarify this issue.  “Rayyan AI software was employed to streamline the organization and screening process to manage duplicates and facilitate title and abstract screening.” | Page 8  Line 216-217 |
| 5. Review the resolution of Figure 1. It is very blurry. | Thank you for pointing this out. We have improved the resolution of Figure 1 for better clarity.  The Figure 1 file has been renamed as Fig1.tif as per journal’s requirement and uploaded in the system accordingly. | Attached as a Fig.1.tif document |
| 6. Mention what other sources were used and mentioned in Figure 1. (n=245). | We appreciate this comment. The other sources, including those from reference lists and grey literature, have been mentioned in the updated Figure 1.  The other sources used were Google Scholar. | Figure 1 (Fig.1) |
| 7. It would be necessary to add in Figure 1, the number of the study that are interventions but do not find the information on motivations and barriers. | Thank you for this suggestion. During the screening phase, our inclusion criteria specifically focused on studies reporting qualitative findings, which resulted in the exclusion of many intervention studies due to their study design. Most of the intervention studies were excluded primarily because they did not meet the qualitative design requirement, rather than for lacking information on motivations and barriers. |  |
| 8. I believe that the review of lifestyle interventions related to exercise, habits and social participation should be limited to not considering the cognitive dimension or only considering the cognitive dimension because they are two different aspects. | We greatly appreciate your thoughtful comment. In response to that, we would like to clarify that the focus of our review is on multidomain interventions that target both physical and cognitive components, as cognitive frailty involves the simultaneous presence of physical frailty and mild cognitive impairment. Separating these dimensions would not provide a complete understanding of how multidomain lifestyle interventions impact this population. We have further clarified this in the revised introduction and discussion section. | Page 4  Line 132-136  Page 38-39  Line 651-655 |
| Results |  |  |
| 9. The selection of interventions is not clear, since there are some studies that are not interventions as such and are considered as interventions, such as cognitive impairment studies (Bechard, et al. 2020; Anna Rosenberg et al., 2020, Haesner et al, 2015). | Thank you for being thorough with our manuscript. We have revised the results and discussion section to better clarify that we included studies which report qualitative findings, some studies explore older adults’ perceptions and experiences regarding lifestyle intervention/modifications rather than testing the interventions directly. This has been clarified to avoid confusion. | Page 11  Line 271-274 |
| 10. The sample size would need to be added in some studies (Bechard). | Thank you for highlighting this. We have updated the manuscript to include sample sizes for the relevant studies.  i | Page 13  Table 3 |
| 11. The proposal of concepts for the analysis of evidence seems unclear because it does not clearly refer to "motivations" but rather to characteristics or conditions that favor the intervention. | Thank you for your insight. In response to that, we have clarified in the manuscript that “motivation” in the context of our study includes both internal and external factors influencing participation. This definition aligns with similar studies that use ‘motivation’ to describe conditions that favor intervention, including what is often referred to as ‘facilitators.’ We hope our clarification is sufficient and agreeable. | Page 10  Line 249-251 |
| 12. The proposal for the analysis of evidence lacks support because concepts such as motivations or conditions that favor the intervention are confused.   Finally, motivation has to do with something individual that these studies do not seem to show and aspects are being sought that these studies do not have. | We have addressed this inquiry in comments number 11 | Page 10  Line 249-251 |
| 13. It is necessary to better work on the objectives of the search and limit it to lifestyle or cognitive interventions. Not to both because it is confusing. | We appreciate your concern. However, since cognitive frailty is characterized by the coexistence of both physical frailty and cognitive impairment, we believe maintaining the inclusion of both lifestyle and cognitive interventions would serve the aims of this review better. Many interventions address both dimensions and separating them would not provide a full understanding of their impact on older adults with cognitive frailty. We have clarified this rationale in the introduction sections. We hope our explanation is sufficient and agreeable. | Page 4  Line 132-136 |
| 14. It is necessary to be clear about what is meant by motivations or to leave them as scopes or conditions that favor the intervention but not as motivations. If we stick with motivations, it is necessary to better explain the concepts and what they are going to refer to in the analysis of the evidence, it is not very clear. | We have addressed this inquiry in comments number 12 and 13. | Page 10  Line 249-251 |
| Academic editor |  |  |
| 1. Please ensure that your manuscript meets PLOS ONE's style requirements, including those for file naming. | Thank you for your reminder. We have revised the whole manuscript to meet PLOS ONE’s requirements. |  |
| 2. We note that the grant information you provided in the ‘Funding Information’ and ‘Financial Disclosure’ sections do not match. When you resubmit, please ensure that you provide the correct grant numbers for the awards you received for your study in the ‘Funding Information’ section. | Noted. Thank you.  We have checked and confirmed the details on funding information.  This study is funded by the Ministry of Higher Education under the Long-Term Research Grant Scheme (LRGS/1/2019/UM-UKM/1/4). |  |
| 3. Thank you for stating the following financial disclosure: "This study is funded by the Ministry of Higher Education under the Long-Term Research Grant Scheme (LRGS/1/2019/UM-UKM/1/4)) and approved by the Research Ethics Committee National University of Malaysia (UKM/PPI/111/8/JEP-2020-34)."   Please state what role the funders took in the study.  If the funders had no role, please state: "The funders had no role in study design, data collection and analysis, decision to publish, or preparation of the manuscript."  If this statement is not correct you must amend it as needed.  Please include this amended Role of Funder statement in your cover letter; we will change the online submission form on your behalf. | "The funders had no role in study design, data collection and analysis, decision to publish, or preparation of the manuscript."  We have included this amended role in the cover letter. Thank you  This study is funded by the Ministry of Higher Education under the Long-Term Research Grant Scheme (LRGS/1/2019/UM-UKM/1/4)) however, the funder funders had no role in study design, data collection and analysis, decision to publish, or preparation of the manuscript. This study also has been approved by the Research Ethics Committee National University of Malaysia (UKM/PPI/111/8/JEP-2020-34). |  |
| 4. If any supporting files for review show as item type ‘other’ please change to item type ‘supporting info’ as the reviewer does not have access to these ’other’ files. | Noted on the names for supporting documents. |  |
| 5. We note that your Data Availability Statement is currently as follows:   "All relevant data are within the manuscript and its Supporting Information files."  Please confirm at this time whether or not your submission contains all raw data required to replicate the results of your study. Authors must share the “minimal data set” for their submission. PLOS defines the minimal data set to consist of the data required to replicate all study findings reported in the article, as well as related metadata and methods | Thank you. Our data availability statement will be as follow;  All extracted data has been included in extraction table and no additional/primary data to be shared as the data are secondary data drawn from already published literature. |  |
